# Dietary and genetic determinants of non-alcoholic fatty liver disease in coronary heart disease patients

**DOI:** 10.1007/s00394-024-03431-w

**Published:** 2024-06-12

**Authors:** Luc Heerkens, Johanna M. Geleijnse, Fränzel J. B. van Duijnhoven

**Affiliations:** Division of Human Nutrition and Health, Wageningen University and Research, Stippeneng 4, P.O. Box 17, 6700 AA Wageningen, The Netherlands

**Keywords:** Non-alcoholic fatty liver disease, Diet quality, Genetic risk, Coronary heart disease patients

## Abstract

**Purpose:**

A healthy diet reduces the risk of non-alcoholic fatty liver disease (NAFLD) in the general population, especially in individuals who are genetically predisposed to NAFLD. Little is known in patients who suffered from a myocardial infarction (MI). We examined the interaction between diet quality and genetic predisposition in relation to NAFLD in post-MI patients.

**Methods:**

We included 3437 post-MI patients from the Alpha Omega Cohort. Diet quality was assessed with adherence to the Dutch Healthy Diet index 2015 (DHD15-index). A weighted genetic risk score (GRS) for NAFLD was computed using 39 genetic variants. NAFLD prevalence was predicted using the Fatty Liver Index. Prevalence ratios (PR) with 95% confidence intervals of DHD15-index and GRS in relation to NAFLD were obtained with multivariable Cox proportional hazards models. The interaction between DHD15-index and GRS in relation to NAFLD was assessed on an additive and multiplicative scale.

**Results:**

Patients had a mean age of 69 (± 5.5) years, 77% was male and 20% had diabetes. The DHD15-index ranged from 28 to 120 with a mean of 73. Patients with higher diet quality were less likely to suffer from NAFLD, with a PR of 0.76 (0.62, 0.92) for the upper vs lower quintile of DHD15-index. No association between the GRS and NAFLD prevalence was found (PR of 0.92 [0.76, 1.11]). No statistically significant interaction between the DHD15-index and GRS was observed.

**Conclusion:**

In Dutch post-MI patients, adherence to the Dutch dietary guidelines was associated with a lower prevalence of NAFLD, as assessed by the FLI. This association was present regardless of genetic predisposition in this older aged cohort.

**Supplementary Information:**

The online version contains supplementary material available at 10.1007/s00394-024-03431-w.

## Introduction

Non-alcoholic fatty liver disease (NAFLD) is considered as the hepatic manifestation of cardiometabolic disorders [1] and has been associated with an increased risk of cardiovascular diseases events in the general population [2] and with an increased cardiovascular mortality risk in drug-treated coronary heart disease (CHD) patients [3]. This latter group is expanding and has a residual risk of cardiovascular mortality despite being medicated, which is most likely attributed to the presence of modifiable cardiometabolic risk factors, such as NAFLD. More evidence on the onset and development of NAFLD is necessary to contribute to better prevention, which can possibly result in an improved prognosis for CHD patients.

The most recommended treatment for NAFLD is weight loss, considering obesity being one of the main drivers [1]. In the general population, evidence suggests that better adherence to dietary guidelines is associated with a lower NAFLD prevalence, as assessed by the Fatty Liver Index (FLI), beyond weight loss [4]. However, previous studies in the general population have utilized dietary scores that are derived from a combination of a limited selection of food groups and nutrients [4, 5]. Dietary scores composed of a broad range of narrowly defined food groups, such as the 2015 Dutch Healthy Diet Index (DHD15-index) are easier to implement in dietary guidelines [6].

NAFLD is not merely a consequence of lifestyle, such as diet, but also depends on genetic predisposition.

However, up until now, the genetic risk for NAFLD is often based on a small number of genetic variants discovered by genome-wide associations studies (GWAS) with a relatively small sample size, explaining only a small fraction of the heritability [7]. Furthermore, some NAFLD-related variants also affect the risk of other cardiometabolic risk factors, such as diabetes [7]. Since NAFLD is a multifactorial disease, a simple genetic risk score (GRS) with only a few NAFLD-related genetic variants without considering their effects on other cardiometabolic risk factors, would discard the complexity of the disease.

The risk of NAFLD by genetic susceptibility can be mitigated by a healthy diet in the general population [5]. The interplay between diet and genetic predisposition, as key determinants of human biology, can provide insight into the onset and development of NAFLD, but this has not been examined in cardiovascular patients before. Therefore, we examined whether diet quality, as assessed by the DHD15-index, and genetic risk, as assessed by a weighted GRS, either alone or combined, are associated with NAFLD prevalence in CHD patients with a history of myocardial infarction (MI) of the Alpha Omega Cohort. In addition, we explored the GRS further by subdividing the score according to the genetic variants’ shared effects on inflammatory and cardiometabolic traits.

## Methods

### Study design and patients

We used data of the Alpha Omega Cohort (NCT03192410), consisting of 4837 drug-treated Dutch patients with a verified history of MI ≤ 10 years prior to study enrolment. Venous blood samples and data on diet, lifestyle, health, medication use, and anthropometrics were collected at the start of the study (i.e. baseline, 2002–2006) [8, 9]. The study was approved by the medical ethics committee of the Haga Hospital (The Hague, the Netherlands) and by the ethics committees of participating hospitals (approval No L01.049). All patients provided verbal and written informed consent for long-term follow-up.

Exclusion of patients for the current study was related to the exposure and outcome data (Supplementary Fig. 1). Hence, the current study excluded patients with missing dietary data (n = 453) as well as patients who had unreliable dietary data (i.e. estimated energy intake of < 800 or > 8000 kcal/d for males; < 600 or > 6000 kcal/d for females; n = 19). Patients with missing genotype data (n = 161) were excluded, which was due to unavailability of blood samples or improper genotyping. We also excluded patients with missing data on BMI (n = 4), waist circumference (n = 21), gamma-glutamyl transferase (GGT, n = 42), and serum triglycerides (n = 61) to calculate the FLI, which we used a predictor of NAFLD. Furthermore, patients with heavy alcohol consumption (males, > 30 g/d; females, > 20 g/d; n = 639) were excluded to rule out the contribution of alcohol consumption to liver fat accumulation.

### DHD15-index

Baseline dietary intake was assessed with a 203-item food frequency questionnaire (FFQ), which was adapted and extended from a validated FFQ on fatty acids and cholesterol intake [10]. Patients were asked to report their usual intake of foods consumed during the previous month; questions on the frequency, amount, and type of foods, as well as preparation methods were included. Trained dietitians checked the returned questionnaires and obtained additional information on unclear or missing items by phone. Quality-assurance procedures included double entry of the FFQ data. Food-consumption data were converted into energy and nutrient intake by using the 2006 Dutch food-composition database [11].

The DHD15-index was created to measure adherence to the Dutch dietary guidelines of 2015 [6]. The DHD15-index consists of 15 components (vegetables, fruit, legumes, nuts, fish, tea, red meat, processed meat, sweetened beverages and fruit juices, sodium, alcohol, fats and oils, dairy, whole grain, and coffee) for which a maximum of 10 points can be obtained for every component, according to the corresponding cut-off or threshold values [6].

### GRS

Venous blood samples (30 mL) were drawn fasted (≥ 8 h, 35% of the analytical sample) or non-fasted and were sent to the laboratory by next-working-day mail service at ambient temperatures. Blood samples were immediately processed into aliquots and stored at -80 degrees Celsius upon arrival [12]. DNA was isolated from buffy coats and genotyping was performed with the Global Screening Array [13]. Genetic variants were imputed to the 1000 Genomes Project reference panel [14]. Genotypes were coded using continuous dosages from 0 to 2. Genetic variants for the GRS were based on the most recent GWAS performed in the Military Veteran Program (MVP) [15], which is a multi-ethnic cohort. This study by Vujkovic et al. [15] observed 77 genetic variants related to NAFLD, of which 55 were also found in European ancestry. Genetic variants in the Alpha Omega Cohort with a minor allele frequency < 0.01 and ambiguous genetic variants were removed, obtaining 39 out of 55 genetic variants from European ancestry for computing the GRS (Supplementary Table 1). A weighted GRS was computed by summing the product of the dosages of the 39 NAFLD-related risk alleles and the corresponding regression coefficients [15]. Subsequently, the weighted GRS was subdivided according to the genetic variants’ effects on other inflammatory and cardiometabolic traits. We obtained sub GRSs with genetic variants related to only NAFLD (n = 9), related to NALFD and inflammatory traits (n = 4), related to NAFLD and cardiometabolic risk factors (n = 7), and related to NAFLD, cardiometabolic risk factors, and inflammatory traits (n = 19) (Supplementary Fig. 2). We also computed a sub GRS with 11 genetic variants that were replicated by the same GWAS of Vujkovic et al. [15], using histology-defined NAFLD.

### Fatty liver index

Baseline FLI was calculated using BMI (kg/m^2^), waist circumference (cm), GGT (U/L), and triglycerides (mg/dL). The score is validated in a Caucasian population-based cohort and is calculated as follows [16]:

FLI = (e^0.953*loge (triglycerides) +0.139*BMI+0.718*loge (GGT) +0.053*waist circumference−15.745^)/(1 + e^0.953*loge (triglycerides) +0.139*BMI+0.718*loge (GGT) +0.053*waist circumference−15.745^) × 100

FLI was categorized into sex-specific tertiles (T1: < 56; T2: ≥ 56- < 79; T3: ≥ 79 for men; T1: < 49; T2: ≥ 49- < 77; T3: ≥ 77 for women), where T3 was used as predictor of NAFLD.

Weight and height were measured at the patients’ home or hospital by trained research nurses, and were used to calculate BMI. Waist circumference was measured at the midpoint between the bottom rib and the top of the hipbone using a non-elastic tape. Serum triglycerides were determined in multiple batches by standard assays (Roche Diagnostics; cat. no. 1488872) on an automated analyzer (Hitachi 912; Roche Diagnostics) with an inter- and intra-assay coefficient of variation < 10%. Serum GGT was determined in one batch after completion of the cohort by standard assays (Abbott Diagnostics; cat. no. 7D6522) on an automated analyser (ARCHITECT ci8200; Abbott) with an intra- and inter-assay CV < 10%.

### Covariates

Data on sociodemographic factors and lifestyle habits were collected through self-administered questionnaires as described in detail elsewhere [8]. Smoking status was categorized into four categories (current; quitted, ≤ 10 years; quitted, > 10 years; never) [17]. Physical activity, presented in metabolic equivalents of tasks (MET), was also categorized into four categories (no; only light (< 3 MET); 0–5 d/week (> 3 MET); > 5 d/week (> 3 MET)) [18].

Liver enzymes (U/L) alanine aminotransferase and aspartate aminotransferase were determined by standard assays (Abbott Diagnostics; cat. No. 8L9222 and 8L9122) on an automated analyzer (ARCHITECT ci8200; Abbott) with an inter- and intra-assay CV < 10% from stored serum samples. Blood lipids (mmol/L, total serum cholesterol and high-density lipoprotein cholesterol [HDL-c]) and plasma glucose (mmol/L) were analyzed using standard kits (Hitachi 912, Roche Diagnostics, Basel, Switzerland). The Friedewald formula was used to calculate low-density lipoprotein cholesterol (LDL-c, mmol/L) [19].

Patients with BMI ≥ 30 kg/m^2^ were classified as having obesity. Diabetes mellitus was considered present in case of a self-reported physician’s diagnosis, use of glucose-lowering medication or elevated plasma glucose (≥ 7.0 mmol/L if fasted > 4 h or ≥ 11.0 mmol/L if not fasted). Systolic blood pressure and diastolic blood pressure were measured twice on the left arm with the patient in a seated position, using an automated device (Omron HEM-711) following a 10-min rest. The values were then averaged.

Self-reported medication use was checked by trained research nurses and coded according to the Anatomical Therapeutic Chemical Classification System [20]: lipid-lowering drugs (C10), antihypertensive drugs (C02, C03, C07, C08, and C09), and glucose-lowering drugs (A10).

### Data analysis

Baseline characteristics are presented across quintiles of DHD15-index as means ± SD for normally distributed data, medians and interquartile range for skewed data, and n (%) for categorical data.

Cox proportional hazards models were used to estimate prevalence ratios (PRs) with 95% confidence intervals (95% CIs) for NAFLD, indicated by sex-specific FLI tertile 3. DHD15-index was analyzed in quintiles, using quintile 1 as the reference category. Model 1 was adjusted for age and sex, and model 2 also included energy intake, physical activity, and smoking status. Confounders were chosen a priori based on literature and were similar to previous studies [4, 5]. Missing data for physical activity (n = 22) were addressed by multiple imputation with 10 iterations and 10 imputations, using the ‘mice’-package in R.

We subsequently performed sensitivity analyses excluding patients with obesity and diabetes, since these cardiometabolic disorders are strongly related to NAFLD. We also examined the continuous association of DHD15-index with NAFLD using restricted cubic splines (RCS), using the fully adjusted model, for the total cohort and for strata of gender. The median of DHD15-index quintile 1 of the total cohort was used as the reference and three knots were placed at 10th, 50th and 90th percentiles according to the Akaike’s information criteria. We also examined the individual DHD15-index components in relation to NAFLD to investigate the individual contributions of the components to the association of DHD15-index with NAFLD. We examined the adherence of the individual components as z-scores (after log transformation, due to skewness of the data), except for coffee. Coffee was analyzed as a binary variable with 0 points to patients who drank coffee and 10 points who did not drink coffee, because no information on brewing methods was available [6].

The overall weighted GRS and all subgroup GRSs were analyzed in categories with the bottom 20th percentile, between the bottom and top 20th percentile, and the top 20th percentile, indicating a low, medium, and high genetic risk for NAFLD, respectively. The third category (high genetic risk) was used as the reference category. The models for all GRSs in relation to NAFLD were adjusted for age, sex and the first ten principal components (PC) for population stratification. Joint effects of DHD15-index and all GRSs in relation to NAFLD were investigated, in which a combined reference category of the lowest DHD15-index quintile and the highest GRS category was chosen. All joint effect models were adjusted for age, sex, energy intake, physical activity, smoking status, and the first ten PCs. Interaction was tested on a multiplicative scale by a product term of DHD15-index and the GRS, and on an additive scale by calculating the relative excess risk due to interaction (RERI), using the upper and lower category of the GRSs and DHD15-index [21].

Two-sided P-values < 0.05 were considered statistically significant. Data analyses were performed using R version 4.2.2 (R Foundation for Statistical Computing).

## Results

Table 1 presents baseline characteristics of the overall patient population (n = 3437) and across quintiles of the DHD15-index. Overall, patients had a mean age of 69 (± 5.5) years, 77% was male and 20% had diabetes. The DHD15-index ranged from 28 to 120 with a mean of 73 (± 13). Patients with the highest diet quality (i.e. quintile 5) were more likely to be female and physically active (> 3 MET) and less likely to be current smokers and to have diabetes compared to patients with the lowest diet quality (i.e. quintile 1).

**Table 1:** Characteristics for the overall population and across quintiles of the DHD15-index in 3437 post-MI patients from the Alpha Omega Cohort, excluding heavy alcohol users

	DHD15-index			
	Total	Q1 (< 75)	Q3 (≥ 83–< 89)	Q5 (≥ 97)
Male	2659 (77.4)	577 (84.0)	511 (74.4)	493 (71.7)
Age, years	69.2 ± 5.5	68.4 ± 5.4	69.3 ± 5.6	69.9 ± 5.4
Physical activity^a^				
No	191 (5.6)	57 (8.3)	34 (5.0)	24 (3.5)
Only light (< 3 MET)	1246 (36.5)	289 (42.1)	239 (35.0)	217 (31.8)
0–5 d/week (> 3 MET)	1255 (36.7)	229 (33.3)	270 (39.6)	266 (39.0)
> 5 d/week (> 3 MET)	723 (21.2)	112 (16.3)	139 (20.4)	175 (25.7)
Smoking status				
Never	625 (18.2)	82 (11.9)	127 (18.5)	150 (21.8)
Former, > 10 years ago	619 (18.0)	104 (15.1)	108 (15.7)	144 (20.9)
Former, ≤ 10 years ago	1640 (47.7)	329 (47.9)	334 (48.6)	333 (48.4)
Current	553 (16.1)	172 (25.0)	118 (17.2)	61 (8.9)
Systolic blood pressure^b^, mmHg	141 ± 21	139 ± 21	143 ± 21	143 ± 21
Diastolic blood pressure^b^, mmHg	80 ± 11	79 ± 11	80 ± 11	80 ± 11
Diabetes	699 (20.3)	135 (19.7)	146 (21.3)	116 (16.9)
Blood parameters				
Plasma glucose^c^, mmol/L	5.58 [5.03–6.58]	5.61 [5.06–6.59]	5.58 [4.99–6.58]	5.48 [5.00–6.28]
LDL cholesterol^d^, mmol/L	2.55 ± 0.82	2.58 ± 0.81	2.59 ± 0.84	2.58 ± 0.79
HDL cholesterol, mmol/L	1.26 ± 0.33	1.25 ± 0.33	1.25 ± 0.31 ±	1.31 ± 0.34
Total cholesterol, mmol/L	4.66 ± 0.95	4.71 ± 0.94	4.71 ± 0.96	4.70 ± 0.93
Alanine transaminase^e^, U/L	16 [13–21]	17 [13–22]	16 [13–21]	16 [13–21]
Aspartate transaminase^f^, U/L	27 [24–32]	28 [24–32]	27 [23–32]	27 [24–32]
AST/ALT ratio^g^	1.79 ± 0.79	1.77 ± 0.85	1.83 ± 1.16	1.78 ± 0.67
Medication use				
Lipid-lowering drugs	2980 (86.7)	570 (83.0)	600 (87.3)	602 (87.5)
Antihypertensive drugs	3089 (89.9)	618 (90.0)	616 (89.7)	608 (88.4)
Antidiabetic drugs	515 (15.0)	87 (12.7)	115 (16.7)	85 (12.4)
Dietary factors				
Energy intake, kcal/day	1875 ± 516	2011 ± 604	1875 ± 517	1782 ± 426
Vegetables, g/d	88 ± 43	77 ± 36	85 ± 39	106 ± 48
Fruit, g/d	111 [45–253]	65 [15–113]	110 [63–242]	236 [111–304]
Wholegrains, g/d	125 ± 59	112 ± 68	126 ± 59	132 ± 50
Legumes, g/d	2 [0–6]	0 [0–3]	2 [0–6]	6 [0–8]
Nuts, g/d	2 [0–3]	1 [0–2]	1 [0–3]	2 [1–7]
Dairy, g/d	282 [172–410]	254 [142- 516]	283 [174- 405]	308 [206- 382]
Fatty fish, serving/week	0 [0–1]	0 [0–0]	0 [0–1]	1 [0–1]
Tea, cups/d	1 [0–4]	0 [0–1]	1 [0–3]	3 [1–4]
Coffee, cups/d	3 [3–5]	3 [3–5]	3 [3–5]	3 [3–5]
Solid fats, g/d	9 [0–22]	19 [8–34]	9 [1–23]	1 [0–6]
Oils, g/d	21 [14–34]	20 [9–32]	22 [13–35]	24 [15–36]
Red meat, g/d	35 [17–51]	43 [21–59]	36 [19–52]	26 [13–44]
Processed meat, g/d	22 [13–45]	42 [19–55]	22 [14–43]	16 [8–24]
Sweetened beverages & fruit juices, cups/d	170 [82–292]	216 [129–375]	170 [81–295]	123 [60–209]
Alcohol, g/d	5 [0–12]	6 [1–15]	5 [0–12]	5 [1–11]
Salt, mg/d	2213 ± 680	2432 ± 809	2187 ± 657	2053 ± 503
FLI components				
BMI, kg/m^2^	27.7 ± 3.8	27.9 ± 3.9	27.6 ± 3.8	27.3 ± 3.6
Waist circumference, cm	102 ± 11	103 ± 11	101 ± 10	100 ± 10
Serum triglycerides, mg/dL	146.14 [107.17–204.60]	150.57 [112.93–207.70]	148.80 [112.48–212.13]	137.28 [100.08–197.73]
Fasting triglycerides^h^, mg/dL	128.43 [96.54–170.94]	131.08 [95.47–165.54]	128.87 [95.66–177.14]	120.46 [94.77–162.53]
Serum GGT, U/L	32 [24–46]	34 [25–51]	31.00 [23–44]	30 [22–43]
Fasting GGT^h^, U/L	32 [24–45]	34 [25–51]	30 [23–44]	30 [23–42]

Values are means ± SDs for normally distributed variables, medians (IQRs) for skewed variables, or n (%) for categorical variables

*DHD15-index* Dutch healthy diet index 2015; *FLI* fatty liver index

^a^Missing data for 22 patients

^b^Missing data for 4 patients

^c^Missing data for 10 patients

^d^Missing data for 163 patients

^e^Missing data for 39 patients

^f^Missing data for 1 patient

^g^Missing data for 40 patients

^h^Missing data for 2281 patients

After multivariable adjustment, a higher DHD15-index was associated with a lower NAFLD prevalence, with a PR (95% CI) of 0.76 (0.62, 0.92) for the upper vs lower DHD15-index quintile (Table 2). This association remained after excluding patients with obesity (PR of 0.74 [0.55, 0.99]) and with diabetes (PR of 0.69 [0.55, 0.88]) (Supplementary Table 2). RCS analyses showed similar results and no differences between male and female patients (Supplementary Fig. 3). When analysing the individual components of the DHD15-index as Z-score in relation to NAFLD, we observed that 1 SD increase of adherence to the dietary guidelines for vegetables, nuts, tea, red meat, processed meat, and sweetened beverages & fruit juices was associated with a lower NAFLD prevalence, while a higher prevalence was observed for legumes (Supplementary Fig. 4).

**Table 2:** Prevalence ratios (95% CI) for DHD15-index (in quintiles) in relation to NAFLD in 3437 post-MI patients of the Alpha Omega Cohort

	DHD15-index		
	Q1 (< 75)	Q3 (≥ 83–< 89)	Q5(≥ 97)
N/cases	687/256	687/237	688/197
Model 1	REF	0.91 (0.77, 1.09)	0.74 (0.61, 0.89)
Model 2	REF	0.93 (0.78, 1.12)	0.76 (0.62, 0.92)

Prevalence ratio (95% confidence interval) obtained from Cox proportional hazards models, using the first quintile of DHD15-index as the reference. NAFLD is predicted using sex-specific FLI tertile 3 (F ≥ 77, M ≥ 79)

Model 1, adjusted for age and sex

Model 2, as model 1 plus energy intake, physical activity, and smoking status

*DHD15-index* Dutch healthy diet index; *FLI* fatty liver index; *NAFLD* non-alcoholic fatty liver disease; *PR* prevalence ratio

Table 3 presents the PRs (95% CI) of genetic risk scores in relation to NAFLD. None of the GRS were associated with NAFLD prevalence. For the total GRS, the PR (95% CI) for the lower vs the upper GRS category was 0.92 (0.76, 1.11). Similar results were found for the sub GRS with shared effects on both inflammatory and cardiometabolic traits (PR of 0.91 [0.76, 1.10]) for the lower vs the upper GRS category. For the other sub GRSs, estimates were more towards the null.

**Table 3:** Prevalence ratios (95% CI) for genetic risk score (in categories) in relation to NAFLD in 3437 post-MI patients of the Alpha Omega Cohort

	Genetic risk score		
	High	Medium	Low
N/cases	688/216	2061/684	688/233
Total (39 genetic variants)	REF	0.99 (0.86, 1.15)	0.92 (0.76, 1.11)
NAFLD only (9 genetic variants)	REF	1.02 (0.87, 1.19)	1.00 (0.83, 1.13)
NAFLD + inflammatory traits (4 genetic variants)	REF	0.97 (0.83, 1.12)	0.96 (0.79, 1.15)
NAFLD + cardiometabolic traits (7 genetic variants)	REF	1.08 (0.93, 1.26)	0.97 (0.80, 1.17)
NAFLD + inflammatory + cardiometabolic traits (19 genetic variants)	REF	0.97 (0.83, 1.12)	0.91 (0.76, 1.10)
Replicated (11 genetic variants)*	REF	1.01 (0.86, 1.17)	0.97 (0.80, 1.17)

Prevalence ratio (95% confidence interval) obtained from Cox proportional hazards models, using the third category of GRS as the reference. NAFLD is predicted using sex-specific FLI tertile 3 (F ≥ 77, M ≥ 79). Model is adjusted for age, sex, and the first ten PCs

*FLI* Fatty liver index; *NAFLD* non-alcoholic fatty liver disease; *PC* principal components; *PR* prevalence ratio

*Genetic variants are replicated using histology-defined NAFLD by Vujkovic et al. [15]

The joint effects of the DHD15-index and genetic risk, using the total GRS, in relation to NAFLD is shown in Table 4. The upper DHD15-index quintile was associated with a lower NAFLD prevalence in all GRS categories, compared to the combined reference category of the lower DHD15-index quintile and upper GRS category. PRs (95% CI) for those in the upper DHD15-index quintile with a high, medium, and low genetic risk were 0.68 [0.45, 1.02], 0.59 [0.43, 0.82], and 0.61 [0.40, 0.92], respectively compared to the combined reference category. The interaction was not statistically significant on a multiplicative scale (P = 0.20), nor on an additive scale (RERI of 0.21 [− 0.39, 0.80]). When examining the joint effects of DHD15-index and the sub GRSs, we also observed that a higher DHD15-index quintile was associated with a lower NAFLD prevalence, in all GRS categories (Table 5). These interactions were also not statistically significant on a multiplicative and additive scale.

**Table 4:** Prevalence ratios (95% CI) for joint effects of DHD15-index and genetic risk score in relation to NAFLD in 3437 post-MI patients of the Alpha Omega Cohort

	DHD15-index			P_multiplicative interaction_	RERI (95% CI)
	Q1	Q3	Q5		
High genetic risk	REF	0.73 (0.49, 1.09)	0.68 (0.45, 1.02)	0.20	0.21 (− 0.39, 0.80)
Medium genetic risk	0.82 (0.60, 1.13)	0.77 (0.56, 1.05)	0.59 (0.43, 0.82)		
Low genetic risk	0.70 (0.48, 1.03)	0.79 (0.53, 1.07)	0.61 (0.40, 0.92)		

Prevalence ratio (95% confidence interval) obtained from Cox proportional hazards models, using a combined category of the lowest DHD15-index quintile and the top 20% of the GRS as the reference. NAFLD is predicted using sex-specific FLI tertile 3 (F ≥ 77, M ≥ 79). P-value for multiplicative interaction is calculated with a product term of DHD15-index and the GRS. The additive interaction is calculated with the RERI (95% CI). Models are adjusted for age, sex, energy intake, physical activity, smoking status, and the first ten PCs

*DHD15-index* Dutch healthy diet index; *FLI* fatty liver index; *NAFLD* non-alcoholic fatty liver disease; *PC* principal components; *PR* prevalence ratio; *RERI* relative excess risk due to interaction

**Table 5:** Prevalence ratios (95% CI) for joint effects of DHD15-index and sub genetic risk scores in relation to NAFLD in 3437 post-MI patients of the Alpha Omega Cohort

	DHD15-index			P_multiplicative interaction_	RERI (95% CI)
	Q1	Q3	Q5		
NAFLD (9 genetic variants)					
High genetic risk	REF	0.95 (0.63, 1.43)	0.77 (0.50, 1.19)	0.44	− 0.11 (− 0.66, 0.43)
Medium genetic risk	1.10 (0.79, 1.52)	0.92 (0.66, 1.28)	0.75 (0.54, 1.06)		
Low genetic risk	0.93 (0.63, 1.38)	0.85 (0.56, 1.29)	0.83 (0.54, 1.26)		
NAFLD + inflammatory traits (4 genetic variants)					
High genetic risk	REF	0.80 (0.54, 1.19)	0.80 (0.53, 1.22)	0.98	− 0.08 (− 0.73, 0.58)
Medium genetic risk	0.86 (0.62, 1.19)	0.83 (0.59, 1.16)	0.61 (0.43, 0.86)		
Low genetic risk	0.83 (0.55, 1.24)	0.76 (0.50, 1.15)	0.63 (0.40, 0.97)		
NAFLD + cardiometabolic traits (7 genetic variants)					
High genetic risk	REF	0.83 (0.55, 1.23)	0.58 (0.37, 0.89)	0.23	0.34 (− 0.19, 0.86)
Medium genetic risk	0.91 (0.66, 1.25)	0.91 (0.66, 1.25)	0.67 (0.48, 0.94)		
Low genetic risk	0.82 (0.55, 1.22)	0.66 (0.44, 1.01)	0.77 (0.51, 1.14)		
NAFLD + inflammatory + cardiometabolic traits (19 genetic variants)					
High genetic risk	REF	0.67 (0.45, 1.00)	0.62 (0.41, 0.93)	0.20	0.33 (− 0.24, 0.89)
Medium genetic risk	0.81 (0.60, 1.10)	0.81 (0.60, 1.09)	0.62 (0.45, 0.85)		
Low genetic risk	0.71 (0.49, 1.04)	0.71 (0.48, 1.06)	0.58 (0.38, 0.87)		
Replicated (11 genetic variants)					
High genetic risk	REF	0.70 (0.47, 1.04)	0.70 (0.47, 1.05)	0.16	0.15 (− 0.41, 0.71)
Medium genetic risk	0.89 (0.66, 1.22)	0.82 (0.60, 1.13)	0.64 (0.46, 0.89)		
Low genetic risk	0.79 (0.53, 1.16)	0.97 (0.66, 1.41)	0.69 (0.46, 0.89)		

Prevalence ratio (95% confidence interval) obtained from Cox proportional hazards models, using a combined category of the lowest DHD15-index quintile and the top 20% of the GRS as the reference. NAFLD is predicted using sex-specific FLI tertile 3 (F ≥ 77, M ≥ 79). P-value for multiplicative interaction is calculated with a product term of DHD15-index and the GRS. The additive interaction is calculated with the RERI (95% CI). Models are adjusted for age, sex, energy intake, physical activity, smoking status, and the first ten PCs

*DHD15-index* Dutch healthy diet index; *FLI* fatty liver index; *NAFLD* non-alcoholic fatty liver disease; *PC* principal components; *PR* prevalence ratio; *RERI* relative excess risk due to interaction

## Discussion

This cross-sectional analysis of 3437 male and female Dutch post-MI patients showed that a higher diet quality was associated with lower NAFLD prevalence, as assessed by the FLI, compared to a lower diet quality. There was no association between genetic risk and NAFLD prevalence, nor was there any interaction between diet quality and genetic risk in relation to NAFLD prevalence. Results were similar for the sub GRS with genetic variants with shared effects on inflammatory and cardiometabolic traits.

Better diet quality, as assessed by adherence to the Dutch dietary guidelines of 2015, was associated with a 24% lower prevalence of NAFLD, as assessed by the FLI, in post-MI patients and was in line with a previous study in the general Dutch population [4]. Rietman et al. examined this relationship vice versa showing that individuals with NAFLD, also assessed by the FLI, had poorer adherence to the Dutch dietary guidelines of 2006. In our post-MI patients, the inverse association between diet quality and NAFLD prevalence can probably be explained by better adherence to the guidelines for limiting sugar sweetened beverages and fruit juices and red and processed meat, which was similar to Rietman et al. [4]. Sugar sweetened beverages and fruit juices contain fructose, which promote liver fat accumulation by exerting de novo lipogenesis [22]. Red meat causes hepatic oxidative stress, which was as shown in animal studies [23]. Processed meat contains heterocyclic amines which are associated with insulin resistance, being a strong risk factor of NAFLD [24], among individuals who were referred to the hospital for a colonoscopy [25]. We also observed that adherence to guidelines for increased intake of vegetables, unsalted nuts, and black and green tea was associated with a lower NAFLD prevalence. Although human studies show inconsistent results, animal studies have shown that black and green tea consumption enhances insulin sensitivity [26]. Fibre, which is present in vegetables and nuts, might promote fibre-degrading gut bacteria, subsequently producing short-chain fatty acids which exert anti-inflammatory effects that act downstream on the liver [27]. Better adherence to the guidelines of legumes, which also contain fibre, was unexpectedly associated with higher NAFLD prevalence in the Alpha Omega Cohort. Legumes are rarely consumed in the Alpha Omega Cohort. About half of the patients in the Alpha Omega Cohort (n = 1818) consume legumes with a median of 6 g/d [4–7]. Possible reasons for this could be that legumes are often consumed as ultra-processed foods or canned, and patients also consume other canned foods, which could be a proxy for unhealthier food choices. Yet, better diet quality overall was associated with a lower NAFLD prevalence in our post-MI patients.

We observed no association in our Dutch post-MI patients between genetic predisposition and NAFLD prevalence, with the total weighted GRS composed of 39 genetic variants, based on a recent GWAS [15]. This study showed that a GRS composed of 77 NAFLD-related genetic variants, with 55 of those originating from European ancestry, was associated with histologically characterized NAFLD in ~ 45,000 multi-ethnic individuals of the general population. Each weighted unit of increase in effect alleles was associated with a log odds increase of 0.60 (0.42, 0.79) [15]. For the sub GRS with shared effects on both inflammatory and cardiometabolic traits, being the largest sub GRS, we observed roughly similar results as with the total GRS in the current study. This was similar with the GWAS of Vujkovic et al. [15], showing a log odds increase of 0.59 (0.40, 0.79) with every weighted unit of increase in the sub GRS with shared effects on both inflammatory and cardiometabolic traits. The PRs for the other sub GRS were smaller than the PR for the total GRS in our study, which was again also similar with the results of Vujkovic et al. [15]. An explanation for the smaller prevalence ratios in our post-MI patients compared to the GWAS of Vujkovic et al. [15] could be the discrepancy in sample size of the Alpha Omega Cohort (n = 3437) and the MVP (n = 218,595) [28] or in the number of genetic variants that constitute the GRS (n = 77 in MVP and n = 39 in Alpha Omega Cohort). Another explanation is the older age of our cohort ranging from 60 to 80 years old, whereas 16.6% of the individuals in the MVP is younger than 60 years and 12.8% younger than 50 years. Age is a well-known effect modifier in the association with GRS and the risk of non-communicable diseases, diminishing the impact of genetic risk when age increases, which may explain the modest result of genetic predisposition in relation to NAFLD in our relatively old post-MI patient population [29]. These findings imply that a genetic predisposition to NAFLD, or to non-communicable diseases in general, may be most relevant in a younger population. Further research on the genetic predisposition of NAFLD is warranted in a younger population.

In the Alpha Omega Cohort, a better diet quality was associated with a lower NAFLD prevalence, regardless of genetic predisposition for NAFLD. Studies examining the interaction between diet quality and genetic predisposition in relation to NAFLD are scarce. A longitudinal study with 2432 middle-aged to older individuals from the Framingham Heart Study did show an interaction between diet quality and genetic predisposition in relation to liver fat accumulation, quantified by CT scans [5]. Ma et al. showed that individuals with a high and medium genetic predisposition accumulated liver fat when their diet quality worsened over time. No difference in liver fat accumulation was observed between different genetic risks in people that maintained or improved their diet quality over time [5]. Since diet quality (data not shown) as well as FLI [3] did not substantially change over time in the Alpha Omega Cohort, we were not able to investigate whether a reduction of diet quality over time is especially worrisome for those post-MI patients with a high genetic susceptibility to NAFLD. Another explanation for the absence of an interaction between diet quality and genetic predisposition could be that with older age the impact of genetic risk diminishes and that exogenous influences, such as diet, have a greater influence on NAFLD. This may imply that a healthy diet is important irrespective of genetic predisposition of NAFLD in older post-MI patients. In younger patients, a healthy diet may be more favourable for NAFLD in those with a higher genetic predisposition compared to lower genetic predisposition, but further research is warranted to study this hypothesis.

The current analysis in this Dutch cohort of post-MI patients with extensive data on blood parameters, anthropometrics, demographics, medication use, diet and lifestyle-related factors had some limitations. The Alpha Omega Cohort comprised older post-MI patients, who were mainly Dutch men. Therefore, our results may be less applicable to female CHD patients and not generalizable to other ancestries and healthy populations, although a similar association between diet quality and the FLI was observed in the general Dutch population [4]. Although the FLI is a validated marker, it is merely a predictor of the presence of NAFLD. Furthermore, in the current study, NAFLD was defined using the FLI with sex-specific tertiles instead of conventional cut-off points. While these tertiles are not validated, they are based on the difference in cardiovascular mortality risk between men and women in the Alpha Omega Cohort [3]. The accuracy of the FLI can be influenced by various factors, such as sex [30], age [31] or BMI [32]. Therefore, evaluation of optimal cut-off points for this specific post-MI population is warranted. Another limitation concerning the observational design of this study includes the inability to infer causality. Furthermore, diet quality and the FLI [3] do not substantially change after 40 months of follow-up and therefore, we are unable to explore the direction of the association between diet quality and the FLI. Finally, we relied on self-reported dietary intake by means of an FFQ, which could have led to systematic underreporting of energy-dense foods or overreporting of healthy foods. However, associations did not change when excluding patients with obesity, who may be more likely to underreport energy-dense foods.

To the best of our knowledge, we were the first to show the association between higher diet quality and lower NAFLD prevalence in CHD patients with a history of MI, using a dietary score that solely consists of well- and narrowly defined food groups. Diet quality is an important determinant of NAFLD prevalence in post-MI patients. However, our results suggest that genetic predisposition to NAFLD does not contribute to this association in a cohort of older post-MI patients. These findings underscore the important role of a healthy diet after MI to lower the risk of NAFLD. Future studies are needed to explore the joint impact of dietary interventions and genetic predisposition on the course of liver fat accumulation in CHD patients across a wide age range.

### Supplementary Information

Below is the link to the electronic supplementary material.

Supplementary file1 (DOCX 245 KB)

## Data Availability

Data described in the manuscript, code book, and analytic code will be made available upon request pending application and approval.

## References

[1] Chalasani N, Younossi Z, Lavine JE, Charlton M, Cusi K, Rinella M, Harrison SA, Brunt EM, Sanyal AJ (2018) The diagnosis and management of nonalcoholic fatty liver disease: practice guidance from the American Association for the Study of Liver Diseases. Hepatology 67(1):328–357. 10.1002/hep.29367 28714183 10.1002/hep.29367

[2] Mantovani A, Csermely A, Petracca G, Beatrice G, Corey KE, Simon TG, Byrne CD, Targher G (2021) Non-alcoholic fatty liver disease and risk of fatal and non-fatal cardiovascular events: an updated systematic review and meta-analysis. Lancet Gastroenterol Hepatol 6(11):903–913. 10.1016/S2468-1253(21)00308-3 34555346 10.1016/S2468-1253(21)00308-3

[3] Heerkens L, van Kleef LA, de Knegt RJ, Voortman T, Geleijnse JM (2023) Fatty liver index and mortality after myocardial infarction: a prospective analysis in the Alpha Omega Cohort. PLoS ONE 18(9):e0287467. 10.1371/journal.pone.0287467 37682815 10.1371/journal.pone.0287467 PMC10490853

[4] Rietman A, Sluik D, Feskens EJM, Kok FJ, Mensink M (2018) Associations between dietary factors and markers of NAFLD in a general Dutch adult population. Eur J Clin Nutr 72(1):117–123. 10.1038/ejcn.2017.148 28901337 10.1038/ejcn.2017.148

[5] Ma J, Hennein R, Liu C, Long MT, Hoffmann U, Jacques PF, Lichtenstein AH, Hu FB, Levy D (2018) Improved diet quality associates with reduction in liver fat, particularly in individuals with high genetic risk Scores for nonalcoholic fatty liver disease. Gastroenterology 155(1):107–117. 10.1053/j.gastro.2018.03.038 29604292 10.1053/j.gastro.2018.03.038 PMC6035111

[6] Looman M, Feskens EJ, de Rijk M, Meijboom S, Biesbroek S, Temme EH, de Vries J, Geelen A (2017) Development and evaluation of the Dutch Healthy Diet index 2015. Public Health Nutr 20(13):2289–2299. 10.1017/s136898001700091x 28625202 10.1017/s136898001700091x PMC10261559

[7] Eslam M, George J (2020) Genetic contributions to NAFLD: leveraging shared genetics to uncover systems biology. Nat Rev Gastroenterol Hepatol 17(1):40–52. 10.1038/s41575-019-0212-0 31641249 10.1038/s41575-019-0212-0

[8] Geleijnse JM, Giltay EJ, Schouten EG, de Goede J, Oude Griep LM, Teitsma-Jansen AM, Katan MB, Kromhout D, Alpha Omega Trial G (2010) Effect of low doses of n-3 fatty acids on cardiovascular diseases in 4,837 post-myocardial infarction patients: design and baseline characteristics of the Alpha Omega Trial. Am Heart J 159(4):539-546 e532. 10.1016/j.ahj.2009.12.033 20362710 10.1016/j.ahj.2009.12.033

[9] Kromhout D, Giltay EJ, Geleijnse JM, Alpha Omega Trial G (2010) n-3 fatty acids and cardiovascular events after myocardial infarction. N Engl J Med 363(21):2015–2026. 10.1056/NEJMoa1003603 20929341 10.1056/NEJMoa1003603

[10] Feunekes GI, Van Staveren WA, De Vries JH, Burema J, Hautvast JG (1993) Relative and biomarker-based validity of a food-frequency questionnaire estimating intake of fats and cholesterol. Am J Clin Nutr 58(4):489–496. 10.1093/ajcn/58.4.489 8379504 10.1093/ajcn/58.4.489

[11] Center NN (2006) Dutch Food Composition Table 2006 NEVO-tabel: Nederlands Voedingsstoffenbestand 2006/NEVO Foundation

[12] Giltay EJ, Geleijnse JM, Schouten EG, Katan MB, Kromhout D (2003) High stability of markers of cardiovascular risk in blood samples. Clin Chem 49(4):652–655. 10.1373/49.4.652 12651820 10.1373/49.4.652

[13] Illumina (2016) Infinium Global Screening Array-24 Kit. Population-scale genetics. https://www.illumina.com/products/by-type/microarray-kits/infinium-global-screening.html. Accessed 25 Jul 2023

[14] Auton A, Brooks LD, Durbin RM, Garrison EP, Kang HM, Korbel JO, Marchini JL, McCarthy S, McVean GA, Abecasis GR (2015) A global reference for human genetic variation. Nature 526(7571):68–74. 10.1038/nature15393 26432245 10.1038/nature15393 PMC4750478

[15] Vujkovic M, Ramdas S, Lorenz KM, Guo X, Darlay R, Cordell HJ, He J, Gindin Y, Chung C, Myers RP, Schneider CV, Park J, Lee KM, Serper M, Carr RM, Kaplan DE, Haas ME, MacLean MT, Witschey WR, Zhu X, Tcheandjieu C, Kember RL, Kranzler HR, Verma A, Giri A, Klarin DM, Sun YV, Huang J, Huffman JE, Creasy KT, Hand NJ, Liu CT, Long MT, Yao J, Budoff M, Tan J, Li X, Lin HJ, Chen YI, Taylor KD, Chang RK, Krauss RM, Vilarinho S, Brancale J, Nielsen JB, Locke AE, Jones MB, Verweij N, Baras A, Reddy KR, Neuschwander-Tetri BA, Schwimmer JB, Sanyal AJ, Chalasani N, Ryan KA, Mitchell BD, Gill D, Wells AD, Manduchi E, Saiman Y, Mahmud N, Miller DR, Reaven PD, Phillips LS, Muralidhar S, DuVall SL, Lee JS, Assimes TL, Pyarajan S, Cho K, Edwards TL, Damrauer SM, Wilson PW, Gaziano JM, O’Donnell CJ, Khera AV, Grant SFA, Brown CD, Tsao PS, Saleheen D, Lotta LA, Bastarache L, Anstee QM, Daly AK, Meigs JB, Rotter JI, Lynch JA, Rader DJ, Voight BF, Chang KM (2022) A multiancestry genome-wide association study of unexplained chronic ALT elevation as a proxy for nonalcoholic fatty liver disease with histological and radiological validation. Nat Genet 54(6):761–771. 10.1038/s41588-022-01078-z 35654975 10.1038/s41588-022-01078-z PMC10024253

[16] Bedogni G, Bellentani S, Miglioli L, Masutti F, Passalacqua M, Castiglione A, Tiribelli C (2006) The fatty liver index: a simple and accurate predictor of hepatic steatosis in the general population. BMC Gastroenterol 6:33. 10.1186/1471-230X-6-33 17081293 10.1186/1471-230X-6-33 PMC1636651

[17] Duncan MS, Freiberg MS, Greevy RA Jr, Kundu S, Vasan RS, Tindle HA (2019) Association of smoking cessation with subsequent risk of cardiovascular disease. JAMA 322(7):642–650. 10.1001/jama.2019.10298 31429895 10.1001/jama.2019.10298 PMC6704757

[18] Haskell WL, Lee IM, Pate RR, Powell KE, Blair SN, Franklin BA, Macera CA, Heath GW, Thompson PD, Bauman A, College A, of Sports M, American Heart A, (2007) Physical activity and public health: updated recommendation for adults from the American College of Sports Medicine and the American Heart Association. Circulation 116(9):1081–1093. 10.1161/CIRCULATIONAHA.107.185649 17671237 10.1161/CIRCULATIONAHA.107.185649

[19] Friedewald WT, Levy RI, Fredrickson DS (1972) Estimation of the concentration of low-density lipoprotein cholesterol in plasma, without use of the preparative ultracentrifuge. Clin Chem 18(6):499–502 4337382 10.1093/clinchem/18.6.499

[20] Nahler G (2009) Anatomical therapeutic chemical classification system (ATC). In: Nahler G (ed) Dictionary of pharmaceutical medicine. Springer Vienna, Vienna, pp 8–8. 10.1007/978-3-211-89836-9_64

[21] Knol MJ, VanderWeele TJ, Groenwold RH, Klungel OH, Rovers MM, Grobbee DE (2011) Estimating measures of interaction on an additive scale for preventive exposures. Eur J Epidemiol 26(6):433–438. 10.1007/s10654-011-9554-9 21344323 10.1007/s10654-011-9554-9 PMC3115067

[22] DiNicolantonio JJ, Subramonian AM, O’Keefe JH (2017) Added fructose as a principal driver of non-alcoholic fatty liver disease: a public health crisis. Open Heart 4(2):e000631. 10.1136/openhrt-2017-000631 29118995 10.1136/openhrt-2017-000631 PMC5663253

[23] Zhu J, Song S, Xu X, Zhou G, Li C (2022) White meat proteins were more conducive to hepatic antioxidative status than soybean and red meat proteins. J Food Biochem 46(4):e13947. 10.1111/jfbc.13947 34561892 10.1111/jfbc.13947

[24] Byrne CD, Targher G (2015) NAFLD: a multisystem disease. J Hepatol 62(1 Suppl):S47-64. 10.1016/j.jhep.2014.12.012 25920090 10.1016/j.jhep.2014.12.012

[25] Zelber-Sagi S, Ivancovsky-Wajcman D, Fliss Isakov N, Webb M, Orenstein D, Shibolet O, Kariv R (2018) High red and processed meat consumption is associated with non-alcoholic fatty liver disease and insulin resistance. J Hepatol 68(6):1239–1246. 10.1016/j.jhep.2018.01.015 29571924 10.1016/j.jhep.2018.01.015

[26] Stote KS, Baer DJ (2008) Tea consumption may improve biomarkers of insulin sensitivity and risk factors for diabetes. J Nutr 138(8):1584S-1588S. 10.1093/jn/138.8.1584S 18641211 10.1093/jn/138.8.1584S

[27] Leung C, Rivera L, Furness JB, Angus PW (2016) The role of the gut microbiota in NAFLD. Nat Rev Gastroenterol Hepatol 13(7):412–425. 10.1038/nrgastro.2016.85 27273168 10.1038/nrgastro.2016.85

[28] Dudbridge F (2013) Power and predictive accuracy of polygenic risk scores. PLoS Genet 9(3):e1003348. 10.1371/journal.pgen.1003348 23555274 10.1371/journal.pgen.1003348 PMC3605113

[29] Jiang X, Holmes C, McVean G (2021) The impact of age on genetic risk for common diseases. PLoS Genet 17(8):e1009723. 10.1371/journal.pgen.1009723 34437535 10.1371/journal.pgen.1009723 PMC8389405

[30] Wu J, Tian S, Li H, Xu Z, Li S, Chen Y-l, Liang X-y, Xiao J, Song J-y, She R-l, Ma C-y, Feng J-h, Li Z-x, Jiang Z-y, Zhang Z-w, Wu K-n, Kong L-q (2022) Population-specific cut-off points of fatty liver index: a study based on the National Health and Nutrition Examination Survey data. BMC Gastroenterol 22(1):265. 10.1186/s12876-022-02303-z 35624410 10.1186/s12876-022-02303-z PMC9145166

[31] Koehler EM, Schouten JN, Hansen BE, Hofman A, Stricker BH, Janssen HL (2013) External validation of the fatty liver index for identifying nonalcoholic fatty liver disease in a population-based study. Clin Gastroenterol Hepatol 11(9):1201–1204. 10.1016/j.cgh.2012.12.031 23353640 10.1016/j.cgh.2012.12.031

[32] Alferink LJM, Darwish Murad S (2019) NAFLD and beneficial effects of lifestyle intervention: defining the meat of the matter. J Hepatol 70(6):1302–1303. 10.1016/j.jhep.2019.01.024 30837092 10.1016/j.jhep.2019.01.024

