# Supplementary material for: Dietary and genetic determinants of non-alcoholic fatty liver disease in coronary heart disease patients
Source: Eur J Nutr. 2024 Jun 12;63(5):1847–56. doi: 10.1007/s00394-024-03431-w (PMC11329394; doi:10.1007/s00394-024-03431-w)
Supplement: Supplementary file 1 — Supplementary file1 (DOCX 245 KB) [file 394_2024_3431_MOESM1_ESM.docx]

**Online Supplementary material**

Title: Dietary and genetic determinants of non-alcoholic fatty liver disease in coronary heart disease patients.

Authors: Heerkens, Geleijnse, Van Duijnhoven

| **Supplementary tables** |  | Page |
| --- | --- | --- |
| Supplementary table 1 | Summary of NAFLD-related genetic variants. | 2 |
| Supplementary table 2 | Prevalence ratios (95% CI) for DHD15 (in quintiles) in relation to NAFLD in post-MI patients of the Alpha Omega Cohort without obesity (n=2623) and without diabetes (n=2738). | 3 |
|  |  |  |
| **Supplementary figures** |  |  |
| Supplementary figure 1 | Flowchart for selection of 3437 post-MI patients of the Alpha Omega Cohort who are available for analysis. | 4 |
| Supplementary figure 2 | Venn diagram presenting overlapping liver (blue), inflammatory (green), and cardiometabolic (red) traits among NAFLD-associated loci. | 5 |
| Supplementary figure 3 | Prevalence Ratios (95% CI) for adherence to DHD15 as continuous exposure in relation to NAFLD in the total cohort (A) and stratified by gender (B) in 3437 post-MI patients of the Alpha Omega Cohort. | 6 |
| Supplementary figure 4 | Prevalence ratios (95% CI) for adherence to individual DHD15 components (as z-scores (except for coffee consumption)) in relation to NAFLD in 3437 post-MI patients of the Alpha Omega Cohort. | 7 |

Supplementary table 1. Summary of NAFLD-related genetic variants.

| rsID | Chr | Position | EA | Effect | MAF | Gene |
| --- | --- | --- | --- | --- | --- | --- |
| rs1337101 | 1 | 219726100 | G | 0.0509 | 0.3 | LYPLAL1;SLC30A10 |
| rs1497406 | 1 | 16505320 | G | 0.0415 | 0.4282 | EPHA2 |
| rs2642438 | 1 | 220970028 | G | 0.0786 | 0.2946 | MTARC1 |
| rs74816838 | 1 | 161643560 | T | 0.0871 | 0.1116 | FCGR2A;FCGR2B |
| rs10195619 | 2 | 112765562 | T | 0.0484 | 0.3908 | MERTK |
| rs10201587 | 2 | 202202791 | A | 0.0454 | 0.4988 | CASP8 |
| rs13409360 | 2 | 113838102 | G | 0.0586 | 0.4052 | IL1RN |
| rs2138157 | 2 | 227103717 | C | 0.064 | 0.356 | IRS1;MIR5702 |
| rs6717858 | 2 | 165539661 | T | 0.0497 | 0.3909 | COBLL1;SCN2A |
| rs7604422 | 2 | 233509761 | A | 0.0529 | 0.3997 | EFHD1 |
| rs4684847 | 3 | 12386337 | C | 0.0725 | 0.1203 | PPARG |
| rs9867368 | 3 | 136147771 | G | 0.0731 | 0.2355 | PCCB |
| rs12500824 | 4 | 77416627 | A | 0.0461 | 0.3503 | SHROOM3 |
| rs71633358 | 4 | 88183817 | T | 0.0835 | 0.2739 | HSD17B13 |
| rs4148824 | 7 | 87075362 | A | 0.0569 | 0.1851 | ABCB4 |
| rs2954038 | 8 | 126507389 | C | 0.1394 | 0.3062 | TRIB1 |
| rs4484649 | 8 | 10571491 | C | 0.0454 | 0.4126 | RP1L1;SOX7 |
| rs4734654 | 8 | 103669991 | A | 0.0517 | 0.362 | KLF10 |
| rs4841133 | 8 | 9183664 | A | 0.1295 | 0.088 | PPP1R3B;TNKS;MFHAS1 |
| rs10883451 | 10 | 101924418 | T | 0.1607 | 0.4766 | ERLIN1 |
| rs2792751 | 10 | 113940329 | T | 0.0752 | 0.2901 | GPAM |
| rs11601507 | 11 | 5701074 | A | 0.0884 | 0.0729 | TRIM5 |
| rs174535 | 11 | 61551356 | T | 0.0606 | 0.3369 | FADS1;FADS2;FADS3 |
| rs7117339 | 11 | 93870338 | C | 0.1283 | 0.1198 | PANX1 |
| rs1169292 | 12 | 121426478 | T | 0.0434 | 0.3914 | P2RX7;HNF1A |
| rs148015593 | 12 | 122523668 | T | 0.0424 | 0.4687 | MLXIP |
| rs4919741 | 12 | 53272920 | G | 0.057 | 0.3415 | KRT84;KRT74 |
| rs11621792 | 14 | 24871926 | T | 0.0422 | 0.453 | NFATC4 |
| rs168144 | 15 | 60914262 | C | 0.0472 | 0.3857 | RORA;ANXA2 |
| rs55868793 | 15 | 73956856 | G | 0.0595 | 0.408 | CD276 |
| rs72754571 | 15 | 90350888 | C | 0.068 | 0.1044 | ANPEP |
| rs112128680 | 16 | 72054052 | A | 0.0498 | 0.2454 | DHODH;HP;HPR |
| rs3810367 | 19 | 4342847 | G | 0.0451 | 0.3763 | SIRT6;STAP2 |
| rs429358 | 19 | 45411941 | T | 0.08 | 0.2221 | APOE;APOC1 |
| rs58542926 | 19 | 19379549 | T | 0.2219 | 0.0749 | TM6SF2 |
| rs7599 | 19 | 36038390 | A | 0.049 | 0.3819 | TMEM147;ATP4A |
| rs2377957 | 20 | 32554473 | A | 0.0473 | 0.5 | AHCY;ITCH |
| rs132665 | 22 | 36564170 | A | 0.0691 | 0.1544 | APOL3 |
| rs1547014 | 22 | 29100711 | C | 0.0642 | 0.3024 | CHEK2 |

Summary statistics are based on GWAS for NAFLD (Vujkovic et al. *Nat Genet.* 2022. 10.1038/s41588-022-01078-z. rsID: reference single-nucleotide polymorphism cluster ID; Chr: chromosome; EA: effect allele; MAF; minor allele frequency.

Supplementary table 2. Prevalence ratios (95% CI) for DHD15 (in quintiles) in relation to NAFLD in post-MI patients of the Alpha Omega Cohort without obesity (n=2623) and without diabetes (n=2738).

|  | DHD15 | | |
| --- | --- | --- | --- |
|  | Q1 | Q3 | Q5 |
| No obesity |  |  |  |
| N/cases | 517/108 | 528/83 | 550/67 |
| Model 1 | REF | 0.88 (0.67, 1.15) | 0.71 (0.53, 0.95) |
| Model 2 | REF | 0.90 (0.68, 1.19) | 0.74 (0.55, 0.99) |
| No diabetes |  |  |  |
| N/cases | 552/180 | 541/154 | 572/125 |
| Model 1 | REF | 0.94 (0.76, 1.16) | 0.67 (0.53, 0.85) |
| Model 2 | REF | 0.95 (0.77, 1.19) | 0.69 (0.55, 0.88) |

Prevalence ratio (95% confidence interval) obtained from Cox proportional hazards models, using the first quintile of DHD15 as the reference. NAFLD is predicted using sex-specific FLI tertile 3 (F ≥ 77, M ≥ 79). DHD15, Dutch Healthy Diet index; FLI, Fatty Liver Index; NAFLD, non-alcoholic fatty liver disease; PR, prevalence ratio.

Model 1, adjusted for age and sex

Model 2, as model 1 plus energy intake, physical activity, and smoking status.


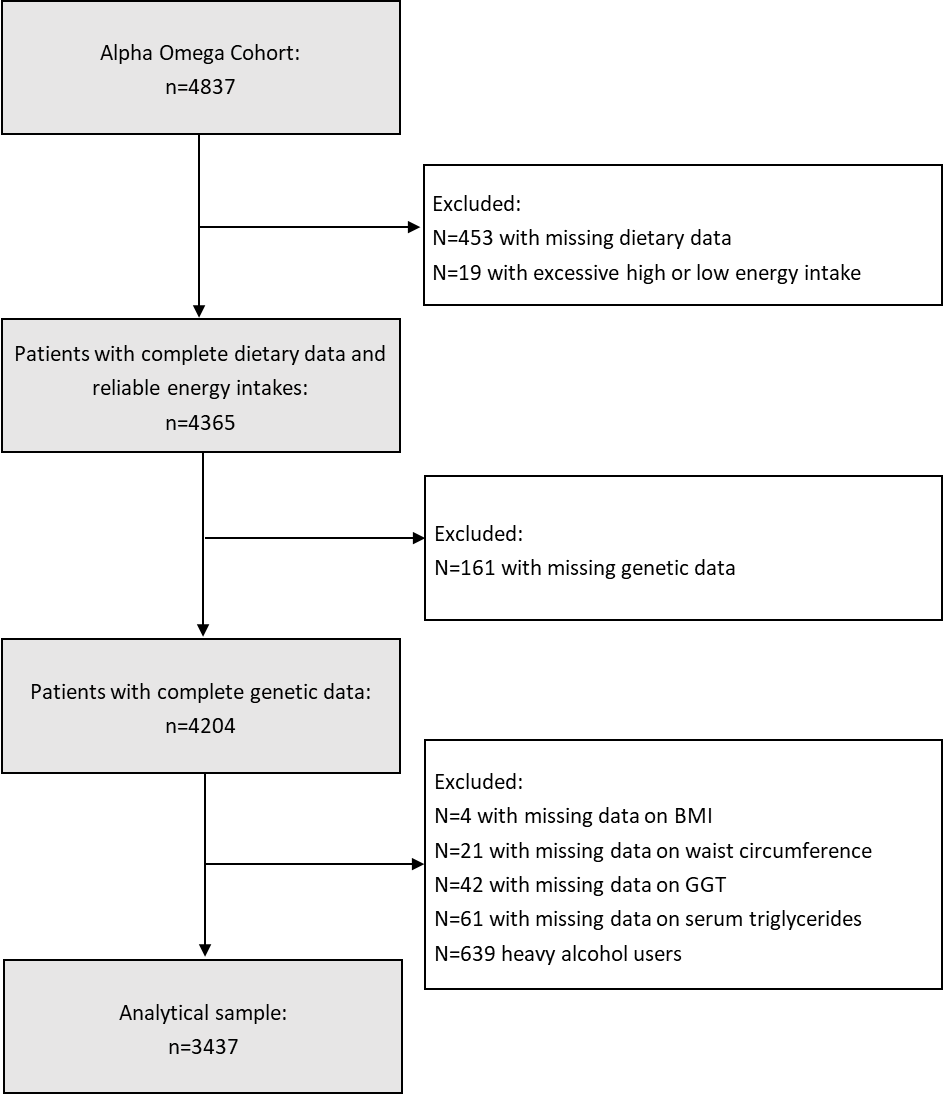


Supplementary figure 1. Flowchart for selection of 3437 post-MI patients of the Alpha Omega Cohort who are available for analysis.
Excessive high or low energy intake is defined as<600 or >6000 kcal/d for females and <800 or >8000 kcal/d for males. BMI, Body mass index; GGT, gamma-glutamyltransferase.


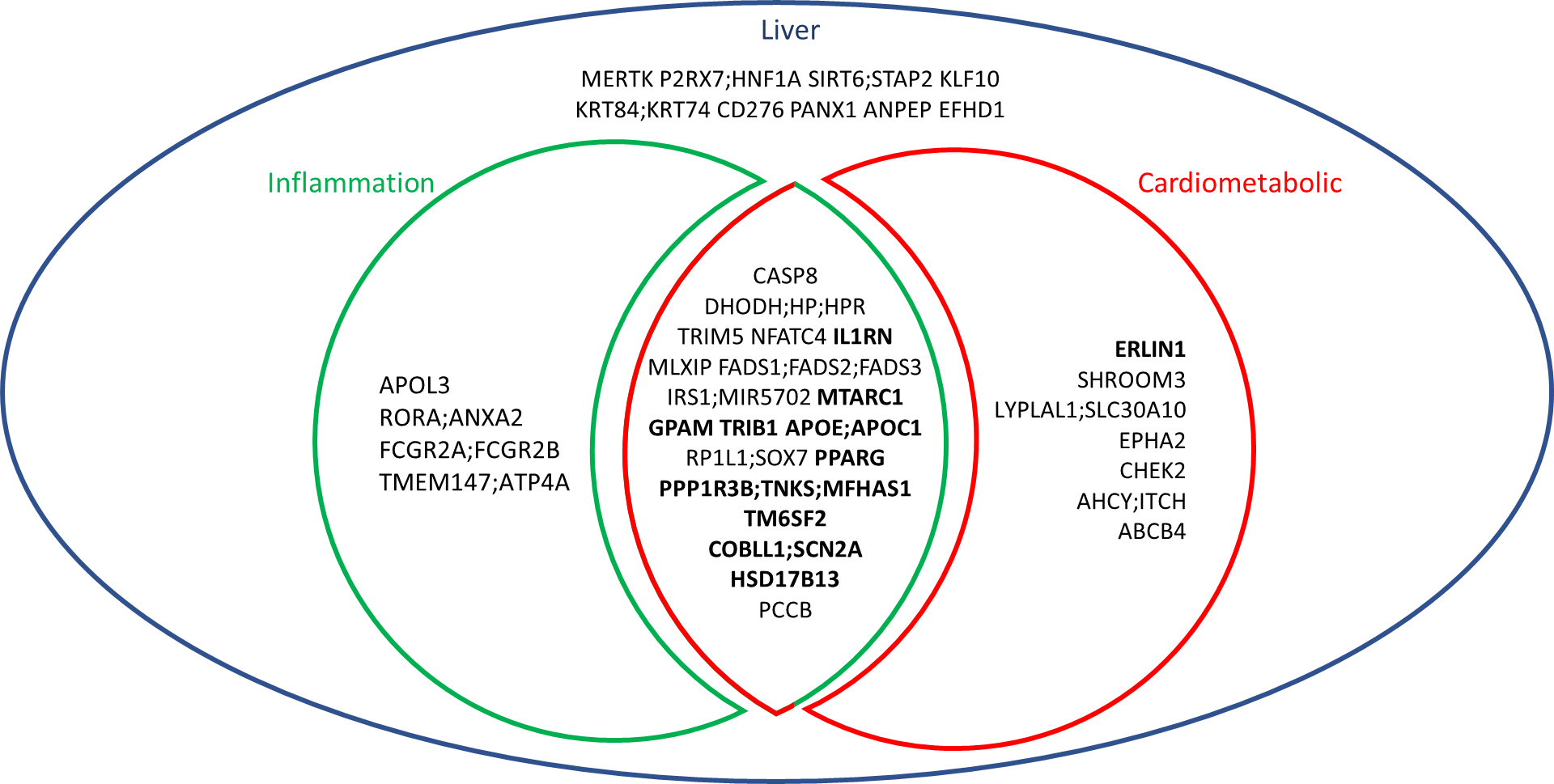


Supplementary figure 2. Venn diagram presenting overlapping liver (blue), inflammatory (green), and cardiometabolic (red) traits among NAFLD-associated loci.
Genetic variant-trait associations are based on the GWAS of Vujkovic et a. (2022). Loci depicted in bold represent replicated genetic variants, also based on the GWAS of Vujkovic et al. (2022).


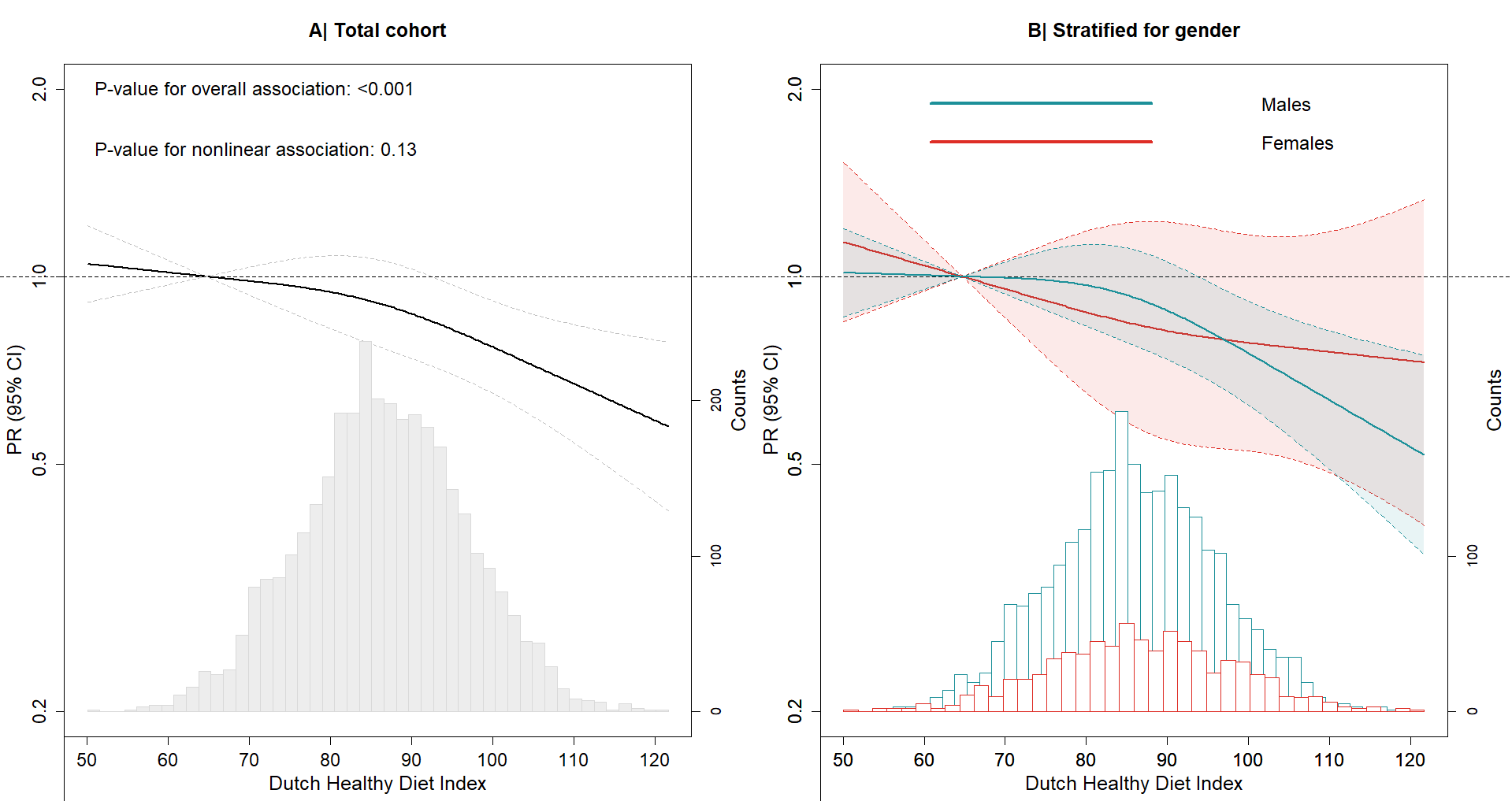


Supplementary figure 3. Prevalence Ratios (95% CI) for adherence to DHD15 as continuous exposure in relation to NAFLD in the total cohort (A) and stratified by gender (B) in 3437 post-MI patients of the Alpha Omega Cohort.
Prevalence ratios with 95% CIs (dotted line) were modeled using restricted cubic splines. Three knots for DHD15 are located at the 10th, 50th, and 90^th^ percentiles. Prevalence ratios are adjusted for age, sex, energy intake, physical activity, and smoking status. NAFLD is predicted using sex-specific FLI tertile 3 (F ≥ 77, M ≥ 79).


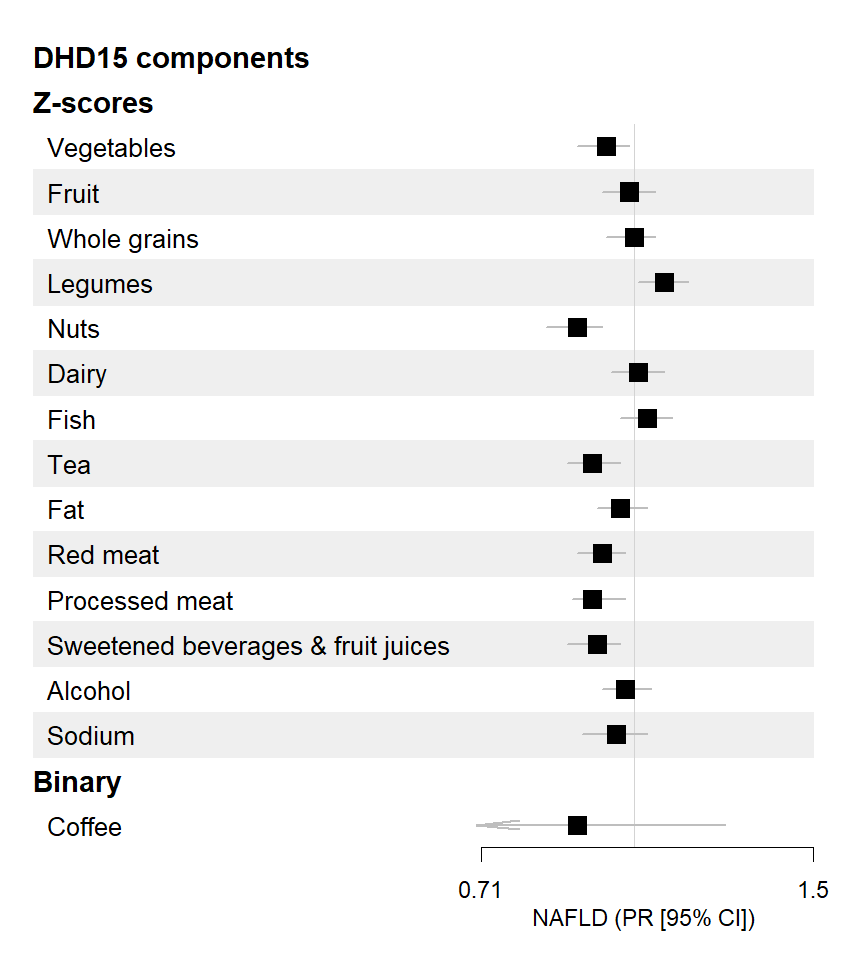


Supplementary figure 4. Prevalence ratios (95% CI) for adherence to individual DHD15 components (as z-scores (except for coffee consumption)) in relation to NAFLD in 3437 post-MI patients of the Alpha Omega Cohort. Prevalence ratios are adjusted for age, sex, energy intake, physical activity, and smoking status. NAFLD is predicted using sex-specific FLI tertile 3 (F ≥ 77, M ≥ 79).
